# Identification of* Streptococcus pneumoniae*: Development of a Standardized Protocol for Optochin Susceptibility Testing Using Total Lab Automation

**DOI:** 10.1155/2017/4174168

**Published:** 2017-03-19

**Authors:** Irene Burckhardt, Jessica Panitz, Florian Burckhardt, Stefan Zimmermann

**Affiliations:** ^1^Department for Infectious Diseases, Microbiology and Hygiene, University Hospital of Heidelberg, Heidelberg, Germany; ^2^EPIET Alumni Network (EAN), Heidelberg, Germany

## Abstract

*Purpose*. Optochin susceptibility is one parameter used in the laboratory to identify* Streptococcus pneumoniae*. However, a single standardized procedure does not exist. Optochin is included neither in the current EUCAST breakpoint tables nor in the CLSI performance standards for antimicrobial susceptibility testing. We wanted to establish an evidence-based protocol for optochin testing for our Total Lab Automation.* Methods*. We tested seven different agars and four different reading time points (7 h, 12 h, 18 h, and 24 h). To accommodate for serotype diversity, all tests were done with 99 different strains covering 34 different serotypes of* S. pneumoniae*. We calculated a multivariable linear regression using data from 5544 inhibition zones.* Results*. Reading was possible for all strains at 12 h. Agar type and manufacturer influenced the size of the inhibition zones by up to 2 mm and they varied considerably depending on serotype (up to 3 mm for serotype 3). Depending on agar and reading time point, up to 38% of inhibition zones were smaller than the cut-off of 14 mm; that is, the result of the test was false-negative.* Conclusions*. Shortening incubation time from 24 h to 12 h for optochin susceptibility testing is feasible. Agar and incubation time have to be chosen carefully to avoid false-negative results.

## 1. Introduction

Even in 2017, the unequivocal identification of* Streptococcus pneumoniae* is still easier said than done. Since its (almost) simultaneous first description in 1881 by Sternberg and Pasteur, several characteristics, which distinguish* S. pneumoniae* from other viridans streptococci, have been described. However, there is no single parameter with 100% sensitivity and specificity. At the beginning of the 20th century, the substance ethylhydrocuprein was described by Morgenroth and Levy (1911) [1]. Today, its derivative ethylhydrocuprein hydrochloride is better known as optochin. Subsequently, it was tested as a treatment option for experimental pneumococcal infections in animals (e.g., for experimental pleuritis in mice) [2]. For reasons of solubility and tolerance, ethylhydrocuprein was never introduced as an i.v. drug for use in humans. For some time, it was used as a local treatment for ocular infections [3]. In the initial trials, ethylhydrocuprein had shown a very specific action on pneumococci, and in 1955 Bowers and Jeffries described the laboratory diagnosis of* S. pneumoniae* using optochin susceptibility and bile solubility [4]. Today, several distributors provide disks with standardized 5 *μ*g optochin content for use in identification of* S. pneumoniae* (e.g., Thermo Scientific, Mast, and Becton Dickinson). Basically this test is an agar diffusion test. However, neither EUCAST nor CLSI included optochin in their guidelines for agar diffusion testing [5, 6]. Published protocols from various sources vary substantially. The Manual of Clinical Microbiology recommends streaking suspicious colonies on a (not further specified) blood agar plate and incubating the test for 18–24 h in 5–7% CO_2_ [7]. One of the disk distributors (BD) recommends using TSA 5% sheep blood agar and incubating it for 24 h or until good growth at 35°C aerobically.

The most recent experimental data on performance of this test is from 1998 [8]. However, since 1998, several aspects of laboratory diagnosis have changed. First, in Europe, EUCAST propagates Müller-Hinton agar with horse blood for susceptibility testing of fastidious organisms (like* S. pneumoniae*), but there are no data on the performance of the optochin disk on Müller-Hinton agar with horse blood. Second, with the advent of full or partial automation in the microbiology laboratory, reading plates after short incubation times (e.g., 12 h) is feasible.

Therefore, we wanted to study several aspects of the diagnosis of* S. pneumoniae* on the TLA using optochin disks. (A) Is it possible to read the plates earlier than 24 h after start of incubation as suggested by the manufacturer of the disks? (B) Does the agar crucially influence the result of optochin susceptibility test and is Müller-Hinton agar with horse blood a suitable agar for optochin susceptibility testing? (C) Taking into consideration the fact that there are more than 90 different serotypes of pneumococci, is there a serotype-dependent influence on the size of the optochin inhibition zone?

## 2. Materials and Methods

For all experiments the following media were used:

Columbia agar with 5% sheep blood (COS): bioMerieux (COS_bmx), Becton Dickinson (COS_BD); Trypticase Soy agar with 5% sheep blood (TSA): bioMerieux (TSA_bmx), Oxoid (TSA_Ox); Müller-Hinton agar with 5% horse blood (MHF): bioMerieux (MHF_bmx), Oxoid (MHF_Ox); Müller-Hinton agar with 5% sheep blood (MHB): bioMerieux (MHB_bmx); all plates used were produced by commercial manufacturers and used within their regular shelf live.

Throughout the manuscript, we use the term agar if we want to refer to a specific agar from a specific manufacturer (e.g., COS_bmx). We use the term agar type if we want to refer to an agar type without a specific manufacturer (e.g., COS).

For all experiments, BD BBL Taxo P Discs were used. They contain 5 *μ*g of optochin each. The cut-off for this disk is 14 mm; that is,* S. pneumoniae* causes diameters of inhibition zones (DOIZ) ≥ 14 mm.

From our large collection of serotyped* S. pneumoniae* strains, we chose 99 strains. Altogether, 34 different serotypes were available. For 31 serotypes, three strains were available, for two serotypes (12A and 33A), only two strains were available, and, for serotype 34, only one strain was available. We also included TIGR4 in our analysis because it is a strain that is widely used in research. For a more detailed description of strains, see Table 1.

All* S. pneumoniae* strains had been confirmed (optochin and bile solubility) and serotyped (Quellung reaction) by the National Reference Center for Streptococci in Aachen, Germany.

All strains were kept at −80°C until usage. They were thawed, inoculated onto a Columbia 5% sheep blood agar (BD), and incubated at 35°C, 5% CO_2_ for 24 hours. One colony was subcultured on COS_BD (35°C, 5% CO_2_, 24 hours). This subculture was used to prepare the respective dilutions for optochin testing.

For standardization purposes, all strains were used in a concentration of 0.5 McF. All plates were streaked, incubated, and imaged using a BD Kiestra Total Lab Automation (TLA) (inoculation volume: 20 *μ*l; streaking pattern 01: sensitivity).

20 *μ*l was chosen as inoculation volume because it resulted (in combination with streaking pattern 01) in confluent growth after 7, 12, 18, and 24 hours of incubation as it is recommended by EUCAST for agar diffusion. Two disks of optochin were added to each plate. For each strain, seven different agar plates were inoculated. DOIZs were determined for two disks per plate at four different points of time. This resulted in (99 × 7 × 2 × 4 =) 5544 measurements altogether. DOIZs were measured and documented using the software tools provided by the manufacturer (ReadA Browser, version 3.0). The system was calibrated each day using reference Petri dishes (RPD) provided by the manufacturer.

Due to the fact that about 10% of all* S. pneumoniae* strains do not grow without an increased CO_2_ level [9], we performed all our experiments with 5% CO_2_.

Each day* S. pneumoniae *ATCC 49619 was tested as a control.

We used Stata Statistical Software Release 12 for descriptive analysis and multivariable linear regression analysis. We modeled the influence of the categorical variables, agar, incubation time, and serotype, on the continuous outcome variable, DOIZ.

## 3. Results

After 7 hours of incubation, 1370 of 1386 DOIZs could be measured. 16 DOIZs could not be determined because of insufficient growth of the respective strain on the plate. Two strains did not grow on MHB_bmx (2x serotype 38), MHF_Ox (2x serotype 38), and TSA_Ox (1x serotype 38 and 1x serotype 6A). One strain did not grow on MHF_bmx and TSA_bmx, respectively (both: serotype 38). After 12 h, 18 h, and 24 h, all strains grew and the expected number of 1386 (99 × 7 × 2) DOIZs for each time point could be determined.

**Figure 1:**
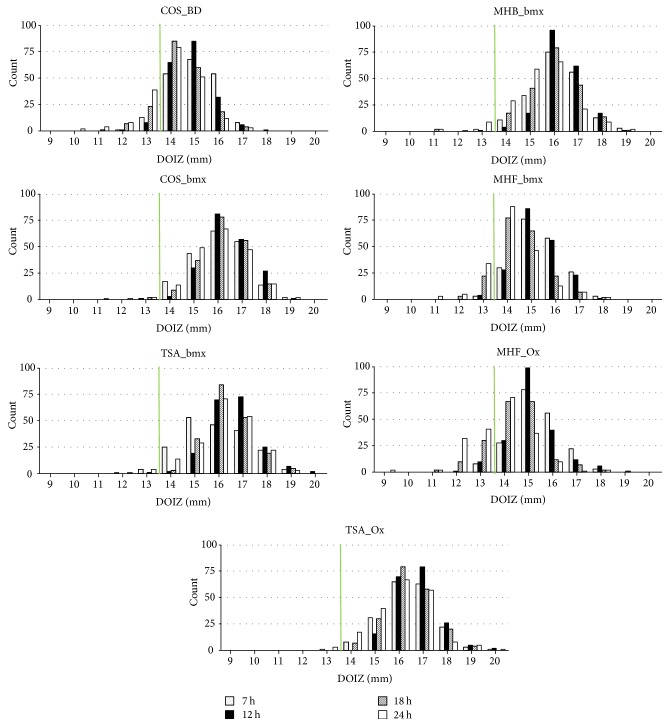
DOIZs (diameters of inhibition zones) by agar (figures) and imaging time point (bars); COS_BD: Columbia agar 5% sheep blood (Becton Dickinson), COS_bmx: Columbia agar 5% sheep blood (bioMerieux), MHB_bmx: Müller-Hinton agar with 5% sheep blood (bioMerieux), MHF_bmx: Müller-Hinton agar fastidious with 5% horse blood (bioMerieux), MHF_Ox: Müller-Hinton agar fastidious with 5% horse blood (Oxoid), TSA_bmx: Tryptic Soy agar with 5% sheep blood (bioMerieux), and TSA_Ox: Tryptic Soy agar with 5% sheep blood; green line: 14 mm cut-off.

DOIZs were measured for all isolates and all agars and varied between 9 mm (24 h, MHF_Ox, serotype 22F) and 20 mm (multiple time points, TSA). For an overview of the distribution of zone sizes for all agars and all time points, see Figure 1. After 7 h of incubation, 1336 of 1370 (97.5%) DOIZs were ≥14 mm. After 12 hours of incubation, 1361 of 1386 (98.2%) DOIZs were ≥14 mm, which was the highest number. After 18 hours of incubation, 1283 (92.6%) DOIZs were ≥14 mm, and after 24 hours of incubation 1190 (85.9%) DOIZs were ≥14 mm, which was the lowest number (Table 2).

Analyzing data according to* agar* revealed that, on TSA_Ox, 788 of 792 (99.5%) DOIZs were ≥14 mm. The agar producing the fewest DOIZs ≥14 mm was MHF_Ox with 654 (82.6%).

Analyzing data according to* agar type* revealed that, on TSA, 1569 of 1584 (99.1%) DOIZs were ≥14 mm. The agar type producing the fewest DOIZs ≥ 14 mm was MHF with 1372 of 1584 (86.6%).

Adding the time of incubation to the analysis revealed that after 12 hours of incubation no agar produced more than 11 DOIZs < 14 mm. After 12 hours of incubation on TSA (TSA_bmx and TSA_Ox) and on COS_bmx, no DOIZ was <14 mm. On MHB_bmx, only one DOIZ was <14 mm. For detailed data on DOIZs and agar categorized into DOIZ < 14 mm and DOIZ ≥ 14 mm, see Table 3.

To statistically evaluate and quantify our results, we calculated a multivariable linear regression using 5528 DOIZs (see Table 4).

For the parameter agar, we chose one of the TSA agars as reference because TSA is the agar type which is recommended by the manufacturer of the optochin disks in the package insert. Of the two TSA agars available, we chose TSA_bmx arbitrarily. For the parameter time, we chose 24 h as reference because that is the incubation time suggested by the manufacturer of the optochin disks in the package insert. For the parameter serotype, we chose serotype 15B as reference because in our experiments it gave the smallest DOIZ.

Comparing the results for the seven agars, the data showed that TSA gave the largest DOIZs. DOIZs for TSA_bmx and TSA_Ox differed by less than 0.1 mm. DOIZs on COS_bmx were only 0.14 mm smaller compared to TSA_bmx, followed by MHB_bmx with DOIZs 0.3 mm smaller. Sizes on MHF_bmx, MHF_Ox, and COS_BD were 1.4 mm, 1.6 mm, and 1.7 mm smaller than on TSA, respectively.

A comparison of the results for the incubation time revealed that DOIZs after 24 h were smallest. Compared to the 24 h values, the DOIZs were 0.3 mm larger after 18 h of incubation, 0.8 mm larger after 12 h of incubation, and 0.6 mm larger after 7 h of incubation.

Analysis of data for the different serotypes showed a maximum of difference in DOIZ with serotype 3. The serotype 3 strains gave DOIZs, which were 2.8 mm larger than DOIZs of serotype 15B strains. 13 serotypes showed a difference in DOIZ of <1 mm. 19 serotypes showed a difference in DOIZ of ≥ 1 mm and <2 mm. Two serotypes showed differences in DOIZ ≥ 2 mm. These were serotype 3 with 2.8 mm difference and serotype 8 with 2.3 mm difference.

## 4. Discussion

Unequivocal identification of* S. pneumoniae* is necessary for adequate patient care and is a prerequisite for accurate epidemiological surveillance of this bacterium. The use of optochin susceptibility for species identification is widely adopted and is a laboratory routine. Therefore, it is surprising that an exact and detailed protocol is still missing. The available protocols leave much room for variations of agar type and incubation time. For all of these parameters, we could show that they substantially influenced the outcome of the optochin susceptibility test.

Large samples like ours favor statistically significant results. It is important to keep in mind which difference in diameter really affects laboratory routine. For our purposes, we decided that a difference of 1 mm or more would make a difference in the lab. Agar choice had the largest influence of almost 2 mm in DOIZ and variation of incubation time could increase DOIZ by almost 1 mm. Looking into our data, the following protocol produced the best results (i.e., the least false-negative results (DOIZ < 14 mm)): TSA (irrespective of manufacturer) or COS_bmx or MHB_bmx and reading time point of 12 h. However, for our routine lab, this protocol holds two challenges: first the agars TSA, COS_bmx, or MHB_bmx and second the 12 h reading time point.

In Europe, using TSA is a problem in terms of availability and workflow. The blood agar commonly used in Europe is not Tryptic Soy but Columbia or MH. Additionally, if we suspect* S. pneumoniae* in a clinical sample, we want to test oxacillin susceptibility directly in parallel to optochin susceptibility. Ideally this can be done on the same plate. However, EUCAST propagates MHF (and not MHB) for susceptibility testing of fastidious organisms, which includes* S. pneumoniae*. Unfortunately MHF produces the smallest DOIZs and the most zones below 14 mm. After 12 hours of incubation, the number of zones <14 mm is still low with *n* = 4 and *n* = 11 for MHF_bmx and MHF_Ox, respectively. It rises to *n* = 42 and *n* = 77 after 24 h of incubation for MHF_bmx and MHF_Ox, respectively. Finally, COS_bmx seems to be a good option for testing optochin susceptibility. However, we realized years ago that the morphology of pneumococci on COS_bmx from patient material is very untypical. They grow very tiny and show no checker-piece appearance after overnight incubation (data not shown).

In terms of an acceleration of the diagnostic procedures, it is reassuring to see that reliable results for optochin testing can be achieved after 12 h and in many cases as early as 7 h. Why DOIZs tend to be smaller after 7 h of incubation than after 12 h of incubation came as a surprise and we cannot explain it completely. We think it is a result of the diffusion kinetics of ethylhydrocuprein hydrochloride in the agar, although we could not find any information on that in the literature. However, reading a plate after 12 h of incubation is difficult in a routine laboratory because this incubation time is normally reached during the middle of the night. 24/7 still is uncommon in most microbiology labs in Germany. However, using Total Lab Automation (TLA) for processing, incubating, imaging, and reading of the plates can be helpful here. With TLA, it is possible to uncouple imaging from reading. One can freely choose the time after which an image is taken. Therefore, imaging after 12 hours is possible and reading can be done as soon as the laboratory starts working again in the morning.

Finally, we would like to point out the fact that serotypes influence the DOIZ even more than incubation time and agar. Other laboratories might have recognized this phenomenon already but to our knowledge it is a still unpublished aspect of pneumococcus identification. Nonetheless, this might have an impact on identification rates of different serotypes. Depending on the identification workflows implemented in the respective laboratories, it is quite possible that certain serotypes are less commonly found because they produce smaller inhibition zones and therefore are more difficult to identify during routine work-up of patient samples.

Our study on optochin susceptibility has two main limitations. First, we used only disks from one manufacturer. It is possible that results with disks from other manufacturers are different. Second, we used McF of 0.5 for all analysis. In our experience, it is rather difficult to produce McF 0.5 solution with suspicious colonies directly from a primary plate. Nevertheless, this study was designed to look for the potential influence of agar, incubation time, and serotype on DOIZ and from our point of view this necessitates this standardization.

As a consequence of our data, we will read our optochin susceptibility plates in the future after 12 h of incubation. As agar, we will use MHF_bmx. This is a compromise between agar availability and workflow necessities. We can use an agar, which is already present in the lab, and we can use one plate for optochin and oxacillin agar diffusion.

## Figures and Tables

**Table 1:** Overview of strains used: species, serotype, number of strains, and origin of strain.

Species	Serotype(s)	Number	Origin
*S. pneumoniae*	19F	1	ATCC 49619
*S. pneumoniae*	4	1	TIGR4 (a gift of R. Hakenbeck, Karlsruhe, Germany)
*S. pneumoniae*	1, 3, 4, 5, 8, 14, 20, 38, 11B, 12F, 15B, 15C, 16F, 17F, 18C, 19F, 22F, 23F, 24F, 35F, 6B, 7F, 9N, 9V, 10A, 11A, 15A, 19A, 23A, 6A, 9A,	3 each	Clinical isolates from invasive pneumococcal disease (IPD) (i.e., septicemia, meningitis, and septic pneumonia)
*S. pneumoniae*	12A, 33A	2 each	Clinical isolates from IPD
*S. pneumoniae*	34	1	Clinical isolate from IPD

**Table 2:** DOIZs (diameters of inhibition zones) stratified by imaging time point and outcome (≥14 mm; <14 mm); bold italic font: least number of DOIZs < 14 mm; ng: no growth.

	7 h	12 h	18 h	24 h
≥14 mm	1336	1361	1283	1190
<14 mm	34	***25***	103	196
ng	16	0	0	0

Total	1386	1386	1386	1386

**Table 3:** DOIZ (diameters of inhibition zones) stratified by agar, imaging time point, and outcome (≥14 mm; <14 mm); COS_BD: Columbia agar 5% sheep blood (Becton Dickinson), COS_bmx: Columbia agar 5% sheep blood (bioMerieux), MHB_bmx: Müller-Hinton agar with 5% sheep blood (bioMerieux), MHF_bmx: Müller-Hinton agar fastidious with 5% horse blood (bioMerieux), MHF_Ox: Müller-Hinton agar fastidious with 5% horse blood (Oxoid), TSA_bmx: Tryptic Soy agar with 5% sheep blood (bioMerieux), and TSA_Ox: Tryptic Soy agar with 5% sheep blood; bold italic font: DOIZ < 14 mm (12 h); ng: no growth.

Agar	DOIZ imaging time point	DOIZ	DOIZ	ng	Total
		<14 mm	≥14 mm		
COS_BD	7 h	14	184	0	**198**
	12 h	***9***	189	0	**198**
	18 h	31	167	0	**198**
	24 h	53	145	0	**198**

COS_BD total		107	685	0	**792**

COS_bmx	7 h	1	197	0	**198**
	12 h	***0***	197	0	**198**
	18 h	2	195	0	**198**
	24 h	4	194	0	**198**

COS_bmx total		9	783	0	**792**

MHB_bmx	7 h	2	192	4	**198**
	12 h	***1***	197	0	**198**
	18 h	2	196	0	**198**
	24 h	12	186	0	**198**

MHB_bmx total		17	771	4	**792**

MHF_bmx	7 h	3	193	2	**198**
	12 h	***4***	194	0	**198**
	18 h	25	173	0	**198**
	24 h	42	156	0	**198**

MHF_bmx total		74	716	2	**792**

MHF_Ox	7 h	8	186	4	**198**
	12 h	***11***	187	0	**198**
	18 h	42	156	0	**198**
	24 h	77	121	0	**198**

MHF_Ox total		138	650	4	**792**

TSA_bmx	7 h	5	191	2	**198**
	12 h	***0***	198	0	**198**
	18 h	1	197	0	**198**
	24 h	5	193	0	**198**

TSA_bmx total		11	779	2	**792**

TSA_Ox	7 h	1	193	4	**198**
	12 h	***0***	198	0	**198**
	18 h	0	198	0	**198**
	24 h	3	195	0	**198**

TSA_Ox total		4	784	4	**792**

Total		**360**	**5168**	**16**	**5544**

**Table 4:** Multivariable linear regression results for agar, imaging time point, and serotype; bold italic font: difference in mm ≥1 mm and <2 mm; bold font: difference in mm ≥2 mm; n.s.: not significant.

	mm difference	*t* statistics	Confidence interval (95%)	*p* value
Agar				
COS_BD	***−1.7***	(−35.58)	[−1.8, −1.6]	<0.001
COS_bmx	−0.1	(−2.71)	[−0.2, −0.0]	<0.01
MHB_bmx	−0.3	(−5.92)	[−0.4, −0.2]	<0.001
MHF_Ox	***−1.6***	(−33.35)	[−1.7, −1.5]	<0.001
MHF_bmx	***−1.4***	(−28.19)	[−1.5, −1.3]	<0.001
TSA_Ox	0.1	(2.05)	[0.0,0.2]	<0.05
TSA_bmx	0.0	(·)	[0.0,0.0]	—
Imaging time point				
7 h	0.6	(17.57)	[0.6,0.7]	<0.001
12 h	0.8	(22.50)	[0.8,0.9]	<0.001
18 h	0.3	(9.25)	[0.3,0.4]	<0.001
24 h	0.0	(·)	[0.0,0.0]	—
Serotype				
1	***1.0***	(9.16)	[0.8,1.2]	<0.001
10A	0.9	(8.20)	[0.7,1.1]	<0.001
11A	***1.5***	(14.08)	[1.3,1.7]	<0.001
11B	***1.0***	(9.33)	[0.8,1.2]	<0.001
12A	0.7	(5.79)	[0.5,0.9]	<0.001
12F	***1.3***	(12.61)	[1.1,1.5]	<0.001
14	***1.0***	(9.27)	[0.8,1.2]	<0.001
15A	***1.5***	(14.02)	[1.3,1.7]	<0.001
15B	0.0	(·)	[0.0,0.0]	—
15C	***1.8***	(16.91)	[1.6,2.0]	<0.001
16F	***1.2***	(11.25)	[1.0,1.4]	<0.001
17F	0.6	(5.99)	[0.4,0.8]	<0.001
18C	***1.9***	(17.98)	[1.7,2.1]	<0.001
19A	***1.8***	(16.85)	[1.6,2.0]	<0.001
19F	0.7	(7.07)	[0.5,1.0]	<0.001
20	***1.1***	(10.52)	[0.9,1.3]	<0.001
22F	0.0	(0.17)	[−0.2,0.2]	n.s.
23A	0.4	(4.01)	[0.2,0.6]	<0.001
23F	0.5	(5.20)	[0.3,0.8]	<0.001
24F	***1.2***	(11.08)	[1.0,1.4]	<0.001
3	**2.8**	(26.69)	[2.6,3.0]	<0.001
33A	***1.0***	(8.14)	[0.7,1.2]	<0.001
34	0.3	(2.00)	[0.0,0.6]	<0.05
35F	0.8	(7.29)	[0.6,1.0]	<0.001
38	***1.3***	(12.27)	[1.1,1.5]	<0.001
4	0.9	(8.26)	[0.7,1.1]	<0.001
5	***1.2***	(11.31)	[1.0,1.4]	<0.001
6A	0.9	(8.09)	[0.6,1.1]	<0.001
6B	***1.5***	(14.59)	[1.3,1.7]	<0.001
7F	0.9	(8.14)	[0.7,1.1]	<0.001
8	**2.3**	(22.16)	[2.1,2.5]	<0.001
9A	***1.4***	(13.68)	[1.2,1.6]	<0.001
9N	***1.1***	(10.29)	[0.9,1.3]	<0.001
9V	***1.4***	(12.83)	[1.1,1.6]	<0.001
TIGR4	0.5	(3.32)	[0.2,0.8]	<0.001
Constant	14.7		[14.5,14.8]	

## Abbreviations

TLA: Total Lab Automation
DOIZ: Diameter of optochin inhibition zone
BD: Becton Dickinson
bmx: bioMerieux
Ox: Oxoid
EUCAST: European Committee on Antimicrobial Susceptibility Testing
CLSI: Clinical and Laboratory Standards Institute
McF: McFarland.
